# The Impact of a Weight Loss Intervention on Diet Quality and Eating Behaviours in People with Obesity and COPD

**DOI:** 10.3390/nu9101147

**Published:** 2017-10-19

**Authors:** Rebecca F. McLoughlin, Vanessa M. McDonald, Peter G. Gibson, Hayley A. Scott, Michael J. Hensley, Lesley MacDonald-Wicks, Lisa G. Wood

**Affiliations:** 1 Priority Research Centre for Healthy Lungs, Hunter Medical Research Institute, University of Newcastle, Callaghan, NSW 2305, Australia; rebecca.zapirain@uon.edu.au (R.F.M.); vanessa.mcdonald@newcastle.edu.au (V.M.M.); peter.gibson@newcastle.edu.au (P.G.G.); hayley.scott@newcastle.edu.au (H.A.S.); michael.hensley@newcastle.edu.au (M.J.H.); 2 Department of Respiratory and Sleep Medicine, John Hunter Hospital, New Lambton, NSW 2305, Australia; 3 Discipline of Nutrition and Dietetics, School of Health Sciences, University of Newcastle, Newcastle, NSW 2308, Australia; lesley.wicks@newcastle.edu.au

**Keywords:** eating behaviours, nutritional biomarkers, fatty acids, carotenoids, COPD

## Abstract

There is a paucity of evidence to guide clinicians about appropriate management strategies for people with obesity and Chronic Obstructive Pulmonary Disease (COPD). We have recently published results from the first weight loss intervention in adults (>18 years) with obesity (body mass index; BMI ≥ 30 kg/m^2^) and COPD, using a low-calorie diet coupled with a partial meal replacement plan and resistance exercise training, which resulted in a 6.4% reduction in weight while maintaining skeletal muscle mass and improving health status. This sub-study aims to evaluate the intervention by (a) examining changes in dietary intake and nutritional biomarkers and (b) examining predictors of weight loss. Dietary intake was evaluated using four-day food diaries, and analysis of plasma fatty acids and plasma carotenoids as biomarkers of dietary fat intake and fruit and vegetable intake, respectively. Twenty-eight obese COPD subjects (*n* = 17 males, *n* = 11 females) with a mean (standard deviation; SD) age of 67.6 (6.3) years completed the 12-week weight loss intervention. Pre-intervention, mean (SD) BMI was 36.3 (4.6) kg/m^2^. Micronutrient intake improved from pre- to post-intervention, with the percentage of subjects meeting the Nutrient Reference Values increased for all micronutrients. Post-intervention, significant decreases in total (*p* = 0.009) and saturated fat intake (*p* = 0.037), and corresponding decreases in total (*p* = 0.007) and saturated plasma fatty acids (*p* = 0.003) were observed. There was a trend towards higher total carotenoids post-intervention (*p* = 0.078). Older age (*p* = 0.025), higher pre-intervention uncontrolled eating (*p* < 0.001) and plasma carotenoids (*p* = 0.009) predicted weight loss. This demonstrates the efficacy of a weight loss intervention in improving diet quality of obese COPD adults.

## 1. Introduction

Chronic Obstructive Pulmonary Disease (COPD) is the third leading cause of mortality worldwide, and it is estimated that approximately 7.5% of Australians aged ≥40 years have disease that is significant enough to cause a daily symptom burden [1]. Although COPD has traditionally been associated with involuntary weight loss, malnutrition and muscle mass depletion [2], the prevalence of obesity (Body Mass Index, BMI ≥ 30 kg/m^2^) in COPD has been reported to be between 18–54% [3,4,5], and is becoming increasingly prevalent particularly in the earlier stages of the disease (Global Initiative for Chronic Obstructive Lung Disease, GOLD stage I and II) [6,7,8,9].

Despite having less severe airflow limitations, people with obesity who have COPD are reported to experience greater exercise intolerance, fatigue and dyspnoea, and poorer health-related quality of life compared to their normal-weight counterparts [10]. These negative consequences of obesity, combined with the increasing prevalence of obesity in COPD, highlight the need for a suitable management approach [11]. There is currently a paucity of evidence to guide clinicians about appropriate management strategies for people with obesity and COPD, and COPD clinical practice guidelines do not address this issue. 

Guidelines for the management of overweight and obesity in the general population recommend that weight loss interventions are designed to create an energy deficit through calorie restriction, increased energy expenditure or a combination of both [12]. The recommendations to assist in achieving reduced caloric intake include reducing consumption of energy dense high-fat foods and replacing them with foods such as fruit and vegetables [12]. Fruit and vegetables are recommended during weight loss, as they are low in energy while being high in micronutrients, such as carotenoids, and high in water and fibre, which contribute to satiety and result in the consumption of fewer calories [13].

Nutrition interventions in COPD to date have focused on malnutrition [14,15]; however, we have recently published results from the first weight loss intervention in people with obesity and COPD. This intervention utilized a low-calorie diet coupled with a partial meal replacement (PMR) plan and resistance exercise training, resulting in a 6.4% reduction in weight while maintaining skeletal muscle mass and improving health status, functional capacity, muscle strength, and biochemical and clinical markers [16]. The impact of this intervention on diet quality, however, has not yet been reported. It is important to examine the impact the intervention had on dietary intake, as poor diet quality can result in adverse health consequences. Examining changes to dietary intake can provide insight into eating habits that are amenable to change and may therefore contribute to the success of the intervention [17]. In addition, we have not previously explored the factors that predicted the likelihood of people with COPD being successful in our weight loss intervention, which is also important to understand, in order to target those individuals most likely to benefit from the intervention.

This sub-study aimed to evaluate our previous weight loss intervention in people with obesity and COPD by (a) examining changes in dietary intake and nutritional biomarkers and (b) examining the clinical, demographic and biochemical predictors of weight loss. We hypothesised that diet quality would improve during the intervention, demonstrated by an increase in fruit and vegetable (F & V) intake and corresponding increase in plasma carotenoids, and a decrease in dietary fat intake, in particular saturated fat, corresponding to a decrease in total and saturated plasma fatty acids. We also hypothesised that subject characteristics including COPD disease severity, age, gender and pre-existing diet quality and eating behaviours would predict the degree of weight loss achieved. 

## 2. Materials and Methods 

### 2.1. Subjects 

A weight loss intervention was conducted in people with obesity and COPD, which has been described in detail previously [16]. Briefly, 33 obese (BMI ≥ 30 kg/m^2^) subjects with COPD (defined by a post bronchodilator forced expiratory volume in one second (FEV_1_) <80% predicted and forced expiratory ratio (FER) <0.70) were recruited. Twenty-eight subjects (*n* = 17 males, *n* = 11 females) with a mean (SD) age of 67.6 (6.3) years completed the intervention. Five subjects were withdrawn from the study (*n* = 2 were too unwell to continue, *n* = 1 was lost to follow up, *n* = 1 withdrew due to abdominal pain and *n* = 1 was withdrawn by investigators due to excessive alcohol intake) [16]. The majority of subjects (57%) had GOLD stage II COPD.

All subjects were ex-smokers with a smoking history of >10 pack years and ceased smoking >6 months prior to study, and had satisfactory English language literacy skills and cognitive function (Mini Mental State Examination score >24). Exclusion criteria included: medical conditions requiring specialised dietary plans, on insulin, change in weight (±5% body weight; BW) in the past three months, presence of orthopaedic problems that may compromise exercise performance, occurrence of an upper or lower respiratory tract infection in the past month, or the presence of an unstable cardiac condition, chronic heart failure or other chronic inflammatory condition that might compromise participation. 

### 2.2. Study Design 

The study was a secondary analysis of a pre and post 12-week weight loss intervention involving a high protein (1.2–1.5 g/kg BW/day), low-calorie diet coupled with a PMR plan (3350–500 kJ/day, based on baseline BMI) [16]. Subjects were instructed to consume 2–3 meal replacements (based on baseline BMI) (Optifast^®^ VLCD™, Nestle Nutrition, Australia; and/or Kicstart™ VLCD, Prima Health Solutions, Frenchs Forest, Australia), one main meal (1200–1750 kJ/day) and self-selected snacks (900–1200 kJ/day) daily. Dietary counselling was undertaken by dietitians experienced in weight loss interventions, with weekly sessions conducted via alternating face-to-face and telephone consults. Dietary counselling included education on energy balance, macronutrients (dietary fat and the different types, carbohydrates and the glycaemic index, and consuming adequate protein (1.2–1.5 g/kg body weight/day)), increasing fruit and vegetable intake, and modifying recipes (i.e., substituting reduced/low-fat varieties for full-fat ingredients, halving the amount of sugar, increasing the amount of vegetables, and removing the skin from chicken and visible fat from meat). Education also provided on food product label reading, with subjects recommended to choose products with less than 10 g total fat per 100 g(mL), less than 10 g sugar per 100 g(mL), and more than 3 g fibre per 100 g(mL). Consults with a dietitian also focused on motivational strategies and personal goal setting for behaviour modification. 

Resistance exercise training was prescribed and supervised by a physiotherapist. This included an individualised home-based strength training program based on the Lung Foundation of Australia recommendations [18]; individuals were encouraged to perform the prescribed exercises on alternate days three to four days per week. Resistance exercise training was chosen over endurance training, as it is an anabolic stimulus, and therefore was incorporated into the intervention to assist in skeletal muscle mass maintenance during weight loss. This research was approved by the Hunter New England Human Research Ethics committee (11/06/15/4.03), and registered with the Australian New Zealand Clinical Trials Registry (ACTRN126000056897). Written informed consent was obtained from all subjects. To maintain confidentiality, personal details were stored separately from the study data, all information was de-identified and a unique study number was generated and allocated to each subject. Only authorized personnel approved under ethics had access to the study data.

### 2.3. Dietary Analysis

Pre- and post-intervention, subjects completed a four-day semi-quantitative food diary. Nutrient analysis was conducted using Foodworks 2009 (version 6, Xyris Nutrient Calculation Software, High Gate Hill, QLD, Australia), and data compared against the Nutrient Reference Values (NRVs) [19]. To examine changes in eating behaviours, the Three-Factor Eating Questionnaire 18-Item (TFEQ-18) [20,21] was also completed pre- and post-intervention. Items in the TFEQ-R18 are divided into three categories; emotional eating, cognitive restraint and uncontrolled eating, with a score out of 100 calculated for each. Higher scores indicate greater emotional eating, cognitive restraint, and uncontrolled eating, respectively. 

### 2.4. Clinical Assessments

Clinical assessments were conducted using validated methodologies as described previously [16]. This included measurement of lung function (post bronchodilator FEV_1_, forced vital capacity (FVC) and FER) by spirometry according to American Thoracic Society (ATS) criteria [22], health-related quality of life (using the St George Respiratory Questionnaire (SGRQ)) [23] and anthropometrics (height, weight, waist circumference, and body composition via dual-energy X-ray absorptiometry (DEXA; GE Lunar Prodigy Pro Dual X-ray Bone Densiometer)). BMI was calculated as weight (kg)/height (m) ^2^. To quantify physical activity levels, the short form of the International Physical Activity Questionnaire (IPAQ) was used [24]. The IPAQ questionnaire assesses the frequency (measured in days per week) and duration (minutes per day) of three specific types of physical activity (walking, moderate-intensity activities and vigorous-intensity activities) [25]. Each type of physical activity is weighted by its energy requirements defined in METS (Metabolic Equivalent of Task; walking = 3.3 METs, moderate physical activity = 4.0 METs and vigorous physical activity = 8.0 METs) and a MET-minutes/week score calculated using the formula “METs × minutes per day × days per week” [24]. Total physical activity MET-minutes/week is the sum of the MET-minutes/week scores for walking, moderate and vigorous physical activity. Using the IPAQ guidelines, a categorical variable for physical activity (low, moderate and high) was also created [24]. 

### 2.5. Nutritional Biomarkers

As the dietary counselling sessions focused on decreasing energy intake by increasing fruit and vegetable intake and decreasing fatty food intake, plasma carotenoids and plasma fatty acids, biomarkers of fruit and vegetables and dietary fat intake respectively, were chosen to assess diet quality. Venous blood samples were collected following a 12 h fast. Plasma was separated from the whole blood and stored at −80 °C until analysis. Gas chromatography (GC) was used to determine plasma fatty acid concentrations. Fatty acids (FAs) were methylated and concentrations determined using the validated method established by Lepage and Roy [26] as described previously [27]. A mixture of methanol/toluene (4:1 *v*/*v*), containing C13:0 and C19:0 and butylated hydroxytoluene (BHT) (0.12 g/L) was added to the plasma sample. FAs were methylated by adding acetyl chloride drop-wise while vortexing and heated to 100 °C for 1 h. The sample was then cooled and 6% K_2_CO_3_ was added to stop the reaction. The sample was centrifuged and the upper toluene layer collected to be used for GC analysis. GC analysis of the FA methyl esters was performed using a 30 m × 0.25 mm (DB-225) fused carbon-silica column, coated with cyanopropylphenyl (J & W Scientific, Folsom, CA, USA). Sample FA methyl ester peaks were identified by comparing their retention times with a standard mixture of FA methyl esters and quantified using a Hewlett Packard 6890 Series Gas Chromatograph with Chemstations software (version A.04.02, Hewlett-Packard, Palo Alto, CA, USA). 

High performance liquid chromatography (HPLC) was used to determine carotenoid (α-carotene, lycopene, β-carotene, β-cryptoxanthin, lutein/zeaxanthin) concentrations in plasma [28], using validated methodology as described previously [29]. A solution comprised of ethanol: ethyl acetate (1:1) containing canthaxanthin was added to the plasma sample and vortexed. The sample was then centrifuged and the supernatant collected. This process was repeated three times, with ethyl acetate added twice to the pellet, followed by hexane. Milli-Q water was added to the pooled supernatant and the mixture was vortexed and centrifuged. After the supernatant was decanted, nitrogen was used to evaporate the solvents. Dichloromethane:methanol (1:2 *v*/*v*) was then added to reconstitute the sample. HPLC was performed on a Hypersil Octadecylsilane (ODS) column (100 mm × 2.1 mm × 5 um) with a flow rate of 0.3 mL/min. A mobile phase of acetonitrile:dichloromethane:methanol 0.05% ammonium acetate (85:10:5 *v*/*v*) and a diode array detector (470 and 297 nm) were used to analyse the carotenoids. Sample carotenoid peaks were identified by comparing their retention times with a standard mixture of carotenoids and quantified using Agilent 1200 Series High Performance Liquid Chromatograph with Chemstations software (Agilent Corporation, Waldbronn, Germany). 

### 2.6. Statistical Analysis 

Change in total plasma carotenoids and fatty acids after the intervention were the primary outcomes for the diet quality analysis. With *n* = 28 subjects, we have 100% power to detect a clinically significant 1 standard deviation (SD) increase in these biomarkers, α = 0.05. Per protocol analyses was conducted using GraphPad Prism Version 5 (GraphPad Software, Inc., La Jolla, CA, USA). Comparison of parametric data was conducted using paired t-tests, reported as mean (SD). Non-parametric data was compared using the Wilcoxon matched-pairs signed rank test, reported as median (interquartile range; IQR). Pre- and post-intervention four-day food diaries were available for 16 of the 28 subjects that completed the intervention. Therefore, statistical analysis of macronutrient and micronutrient dietary intake data is based on 57% of the study subjects. The Mann Whitney test was used to examine differences between subjects with and without pre- and post-intervention four-day food diaries, in regards to baseline characteristics and outcomes including weight loss, BMI, eating behaviours, plasma fatty acids and plasma carotenoids. Minitab Version 13 (Minitab Statistical Software, Minitab Inc., State College, PA, USA) was used to perform backward stepwise multiple linear regression analysis to examine predictors of weight loss success. Percentage of weight change was used as the outcome of multiple linear regression models. Correlation analysis was conducted to identify variables that correlated with % weight change. Variables with a *p*-value ≤ 0.2 were entered into the model. Differences were considered statistically significant at *p* < 0.05. 

## 3. Results

Subject characteristics and clinical changes following the intervention have been reported previously [16]. Baseline characteristics are presented in Table 1. Briefly, the intervention achieved a weight loss of 6.4% and a significant reduction in mean BMI from 36.3 (4.6) to 33.6 (4.4) kg/m^2^ (*p* < 0.0001). Waist circumference was reduced by a mean (SD) of 8.0 (0.7) cm (*p* < 0.0001). Pre-intervention 57% of subjects had a ‘low’ physical activity level, 26% had a ‘moderate’ physical activity level and 17% had a ‘high’ physical activity level. Total physical activity significantly increased from 617 (99–2118) MET minutes/week pre-intervention to 1685 (1013–3701) MET minutes/week post-intervention (*p* = 0.0446). Subsequently, the percentage of subjects with ‘low’ physical activity levels decreased to 14%, with 55% of subjects classified as having ‘moderate’ and 32% classified as having ‘high’ physical activity levels. 

**Figure 1:**
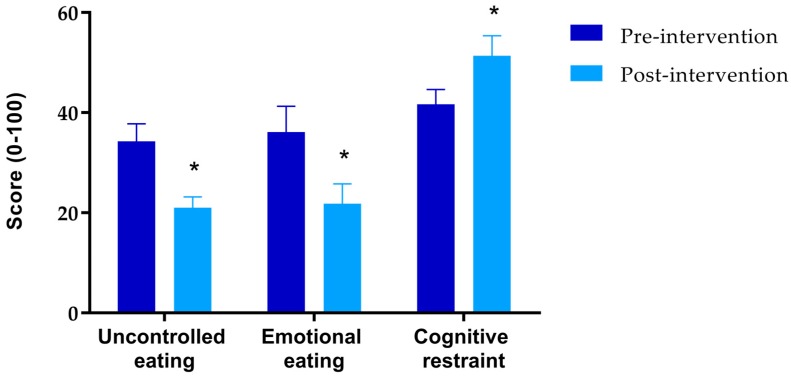
Effect of the weight loss intervention on uncontrolled eating, emotional eating and cognitive restraint measured by the Three-Factor Eating Questionnaire 18-item. * *p* < 0.05.

Significant decreases in both uncontrolled and emotional eating and a significant increase in cognitive restraint were observed following the intervention (Figure 1). Correlation analysis demonstrated that a greater decrease in uncontrolled eating from pre- to post-intervention was associated with greater % weight loss (*r* = 0.453, *p* = 0.016). Changes in cognitive restraint and emotional eating were not associated with % weight change (*p* = 0.414 and *p* = 0.368, respectively).

Pre- and post- intervention four-day food diaries were available for sixteen subjects, with estimated macronutrient intakes presented in Table 2. There were no significant differences in pre- and post-intervention outcomes including weight, BMI, eating behaviours, plasma fatty acids and plasma carotenoids, between these sixteen subjects and the twelve subjects that did not have pre- and post-intervention food diaries. 

Sixty-nine percent of these subjects consumed an average of two meal replacements per day, and therefore complied with the PMR component of the intervention. Post-intervention, there was a significant decrease in total dietary energy intake, with a change in the percentage of energy contributed by each macronutrient. Mean (SD) protein intake increased from 0.9 (0.1) to 1.2 (0.3) g/kg BW (*p =* 0.0002). Micronutrient intake improved from pre- to post-intervention (Table 3), with the percentage of subjects meeting the NRVs increased for all micronutrients [19]. 

Following the intervention, there was a significant decrease in total plasma fatty acids, saturated fatty acids (SFA), monounsaturated fatty acids (MUFA), omega-6 polyunsaturated fatty acids (PUFA) and the omega-6 PUFA: omega-3 PUFA ratio (Table 4). Plasma α- and β- carotene significantly increased (Table 4). 

Using multiple regression analysis, older age, higher pre-intervention total plasma carotenoids and higher pre-intervention uncontrolled eating were identified as predictors of % weight loss (Table 5). Gender and pre-intervention GOLD stage were not found to be predictors of weight loss success, and similarly, COPD disease severity (as per GOLD staging) was not correlated with % weight change (*r* = −0.109, *p* = 0.581). There were also no significant correlations between pre-intervention total physical activity (MET-min/week) or change in total physical activity (MET-min/week), and % weight change (*p* = 0.475 and *p* = 0.212 respectively). 

## 4. Discussion

This study demonstrates for the first time the feasibility and efficacy of a weight loss intervention in reducing energy intake, and improving eating behaviours and dietary quality, particularly in regards to dietary fat and micronutrient intake, in people with obesity and COPD. The findings of this study suggest that older age, higher pre-intervention plasma carotenoid levels and uncontrolled eating behaviour predict greater weight loss success. 

Several components of the intervention are likely contributors to the improvements in dietary quality observed. Studies have shown that the use of a PMR plan is associated with greater nutritional adequacy and dietary adherence [30,31,32] compared to a traditional diet-only approach to weight loss. Of the 16 subjects that completed and returned their post-intervention four-day food diaries, 69% were adherent with the PMR plan. Furthermore, the results of the four-day food diaries and nutritional biomarker analysis demonstrated improvements in overall dietary intake. This is likely to be, at least in part, the result of the dietetic counselling and education provided to the subjects about reducing intake of fatty foods and increasing F & Vs in their diet. 

As hypothesized, we observed a decrease in total and saturated fat intake, and corresponding plasma fatty acid concentrations during the intervention. Consequently, the percentage of dietary energy from total fat also decreased, meeting the Acceptable Macronutrient Distribution Range (AMDR) recommendations (20–35% total energy) [19]. Saturated fat intake remained above the AMDR of <10% total energy [19], although this is likely to be an effect of the high protein component of the diet, with protein food sources such as meat and dairy products often high in saturated fat. Nonetheless, plasma fatty acid analysis demonstrated a significant improvement in the omega-6:omega-3 ratio. This is promising, as a lower ratio is more desirable in reducing inflammation and the risk of chronic diseases [33]. 

Weight loss and exercise are both associated with improvements in insulin sensitivity [34], which could subsequently increase insulin-stimulated suppression of adipocyte lipolysis [35]. Therefore, it is possible that the reduction in circulating plasma fatty acids observed was not the result of reduced dietary fat intake alone, with the effect of weight loss and exercise also possible contributors. 

Our results also demonstrate a trend towards increased total plasma carotenoids during the intervention, with a significant increase in α- and β-carotene observed. Although dietary β-carotene did not significantly increase, it is important to consider that four-day food diaries were only available for 57% of subjects and that there was large variability between these individuals as evidenced by the wide interquartile ranges. Nonetheless, examination of the available four-day food diaries indicated that subjects increased their intake of orange, red and yellow fruits and vegetables such as oranges, carrots, red peppers and tomatoes, which are rich sources of α- and β-carotene [36]. This is promising as increased F & V consumption and subsequently plasma carotenoid levels have been shown to have positive effects on lung function in COPD, which is at least in part due to their antioxidant properties [37,38].

Changes to eating behaviours may have also played a role in improving diet quality. We identified a significant decrease in uncontrolled and emotional eating, and increase in cognitive restraint during the intervention. Uncontrolled eating behaviours have been associated with a high consumption of energy-dense [20], salty and fatty foods [39], and emotional eating behaviours have been associated with a high consumption of energy-dense, sweet and/or fatty foods [20,39]. In contrast, cognitive restraint is typically characterised by a diet lower in energy and fat dense foods and higher in healthy food groups including F & Vs [20]. Changes in eating behaviours observed during the intervention are reflected by the improvements in dietary quality identified from nutrient intake and biomarker analyses that we observed. 

A significant increase in protein intake was also observed following the intervention. Considering the evidence that muscle mass depletion in COPD is a risk factor for mortality [40], muscle mass maintenance during weight loss is particularly important in this population. The recommended protein intake for the general population is 0.75–1.0 g/kg BW/day [19], with higher intakes recommended for muscle maintenance during diet-induced weight loss interventions [41]. Increased protein intake to 1.2 (0.3) g/kg BW, combined with the resistance exercise regimen, are likely to have contributed to the maintenance of skeletal muscle mass during the intervention [16]. These results highlight the benefits, acceptability, and efficacy of incorporating a high protein diet and resistance exercise regime into a weight loss intervention for this population. 

Factors predicting weight loss success during the intervention were also investigated. Older age was identified as a predictor of weight loss success. This finding is consistent with other studies [42,43], and is often suggested to be due to greater intervention adherence in older individuals who have increased concern about their future health, and/or unintentional age-related weight loss [43,44]. Skeletal muscle mass depletion that occurs with age is also likely to be accelerated during a weight loss intervention [45], contributing to a greater overall weight loss. However, this weight loss intervention was found to be effective in maintaining skeletal muscle mass [16].

Higher pre-intervention uncontrolled eating was also identified as a predictor of weight loss success. Uncontrolled eating behaviours are commonly associated with increased body weight and obesity [46,47,48] and, in contrast to our results, Keranen et al. [49] reported an association between higher pre-intervention uncontrolled eating and weight loss failure. In our study, we found that subjects with higher uncontrolled eating pre-intervention were more successful at achieving weight loss as they were able to achieve a greater decrease in uncontrolled eating. This is likely to have been associated with a greater decrease in the consumption of energy and fat dense food [20], which is conducive to weight loss. During the intervention, a dietitian provided dietary counselling that targeted eating behaviours. These findings suggest that the counselling was effective, and highlights the importance of dietetic input in weight loss interventions for this population. In regards to plasma carotenoids as a predictor of weight loss, as they are a nutritional marker for intake, this may indicate that subjects with a higher pre-intervention intake of F & V may have found the dietary intervention more acceptable and thus had better adherence. 

Although total physical activity (MET-min/week), as determined using the IPAQ, significantly increased from pre- to post-intervention, pre-intervention total physical activity and change in total physical activity were not significantly correlated with % weight change. While a resistance exercise training regime was incorporated in the weight loss intervention, this aimed to assist in skeletal muscle mass maintenance during weight loss and was not targeted at achieving weight loss. 

A potential limitation of this study is that a control group was not included in the study design, which can result in an overestimate of the effect size of the intervention. However, the weight loss intervention was intended as a proof of concept study and therefore provides useful information and direction for a larger and more rigorous randomised controlled trial in the future. Another limitation was that only 57% of subjects returned both their pre- and post- intervention four-day food diaries. As a result, we were only able to determine adherence to the PMR of these subjects, limiting our ability to assess adherence as a predictor of weight loss success in the multiple regression analysis. It is possible that this data was not missing at random, with those who were non-compliant with the intervention failing to return their four-day food diaries. Therefore, there is a possibility that the missing data may introduce bias. However, as there were no statistical differences in pre- or post-intervention outcomes (including weight, plasma carotenoids, plasma fatty acids, and eating behaviours) between subjects who did have pre- and post-intervention four-day food diaries and subjects who did not, this is unlikely. 

It is also important to consider that misreporting of dietary intake is common particularly in the obese population [50]. Therefore, our study was strengthened by the use of nutritional biomarkers, with data available for 100% of subjects, to examine changes in dietary intake. By objectively confirming changes in dietary fat intake and fruit and vegetable intake using nutritional biomarkers, we were able to objectively determine that changes in dietary quality were achieved by the intervention. 

## 5. Conclusions

Given the increasing prevalence of obesity in the COPD population, evidence to aid the development of clinical practice guidelines is urgently needed. We have shown for the first time that, in addition to inducing weight loss and improving COPD clinical outcomes [16], a low-calorie, high-protein diet coupled with a PMR plan and dietetic consultations has the potential to improve the dietary quality and eating behaviours of obese adults with COPD. Furthermore, muscle mass maintenance is critical for individuals with COPD, as loss of skeletal muscle mass is a short-term risk factor for mortality [40]. Weight loss, particularly in older adults is often accompanied by loss of muscle mass [51]; however, this novel intervention was successful in achieving muscle mass maintenance through the incorporation of resistance exercise training and increasing protein intake, while not compromising overall dietary quality. Overall, these findings suggest that weight loss strategies and dietary recommendations for weight loss used in the general population may also be effective in the obese COPD population. Further research is needed to examine the long-term efficacy of this weight loss intervention in people with obesity and COPD, in order to enable better clinical management of this population and assist in the development of dietetic practice guidelines. 

## Figures and Tables

**Table 1:** Subject characteristics at baseline (*n* = 28).

Characteristics	Number or Mean (SD)/Median (IQR)
Gender M|F	17│11
Age (years), mean (SD)	67.6 (6.3)
BMI (kg/m^2^), mean (SD)	36.3 (4.6)
Weight (kg), median (IQR)	95.1 (87.1–110.3)
Waist circumference (cm), mean (SD)	121.7 (10.9)
%Body fat, mean (SD)	45.2 (6.3)
Health status—SGRQ, mean (SD)	52 (15)
Post BD FEV1 %predicted, mean (SD)	61.6 (17.1)
Post BD FVC %predicted, mean (SD)	78.6 (20.3)
Post BD FER, mean (SD)	61.7 (12.6)
GOLD Stage I/II/III/IV (*n*)	5/16/7/0
Total physical activity (MET-mins/week)	617 (99–2118)

M, male; F, female; SD, standard deviation; BMI, Body Mass Index; IQR, Interquartile Range (25th–75th centiles); SGRQ, St George Respiratory Questionnaire; Post BD FEV_1_, Post bronchodilator Forced Expiratory Volume in one second; Post BD FVC, Post bronchodilator Forced Vital Capacity; Post BD FER, Post bronchodilator forced expiratory ratio; GOLD, Global initiative for chronic obstructive lung disease; MET, multiples of the resting metabolic rate.

**Table 2:** Intake of selected macronutrients before and after the weight loss intervention, as determined by self-reported four-day semi-quantitative food diaries. (*n* = 16).

	Pre-Intervention (*n* = 16)	Post-Intervention (*n* = 16)	*p*-Value
Energy (kJ), mean (SD)	7101 (1509)	6045 (1247)	0.0007
Total fat (g), mean (SD)	62 (20)	47 (14)	0.0086
SFA (g), mean (SD)	25 (11)	19 (5)	0.0366
PUFA (g), mean (SD)	10 (4)	7 (3)	0.0095
MUFA (g), median (IQR)	24 (17–25)	15 (12–19)	0.0121
Carbohydrate (g), mean (SD)	176 (53)	131 (39)	0.0001
Protein (g), mean (SD)	85 (14)	107 (19)	0.0004
Fibre (g), median (IQR)	20 (19–27)	22 (21–28)	0.2447
% Energy from each macronutrient			
% Total fat, mean (SD)	32 (7)	28 (4)	0.0542
% SFA, mean (SD)	13 (4)	12 (3)	0.4123
% Carbohydrate, median (IQR)	38 (37–43)	35 (32–38)	0.0140
% Protein, median (IQR)	20 (18–23)	31 (27–33)	0.0005

SD, standard deviation; IQR, Interquartile Range (25th–75th centiles); SFA, saturated fatty acid; MUFA, monounsaturated fatty acid; PUFA, polyunsaturated fatty acid; % represents the proportion of dietary energy provided by the specified nutrient. Statistical significance defined as *p* < 0.05.

**Table 3:** Intake of selected micronutrients before and after the weight loss intervention, as determined by self-reported four-day semi-quantitative food diaries. (*n* = 16).

	Pre-Intervention (*n* = 16)		Post-Intervention (*n* = 16)		*p*-Value
		% of Subjects Meeting RDI(AI) *		% of Subjects Meeting RDI(AI) *	
Retinol (µg), median (IQR)	296.0 (150.0–398.0)	6.3	567.0 (496.0–727)	18.8	0.0090
β-carotene (µg), median (IQR)	2468.0 (1250.0–4172.0)	N/A	2369.0 (2157.0–5903.0)	N/A	0.4851
Thiamin (mg), median (IQR)	1.0 (1.0–2.0)	68.8	2.0 (2.0–3.0)	93.8	0.0214
Riboflavin (mg), median (IQR)	2.0 (1.0–2.0)	68.8	3.0 (2.0–4.0)	100.0	0.0006
Niacin equivalent (mg), median (IQR)	42.0 (37.0–44.0)	100.0	45.0 (41.0–52.0)	100.0	0.1272
Vitamin C (mg), median (IQR)	66.0 (50.0–93.0)	87.5	120.0 (86.0–151.0)	93.8	0.0591
Vitamin D ( µg), mean (SD)	4.0 (2.0)	(0.0)	10.0 (3.0)	(43.8)	<0.0001
Vitamin E (mg), median (IQR)	7.0 (5.0–9.0)	(25.0)	15.0 (12.0–17.0)	(81.3)	0.0012
Sodium (mg), mean (SD)	2266.0 (587.0)	43.8	1988.0 (739.0)	31.3	0.1386
Potassium (mg), median (IQR)	2670.0 (2326.0–3193.0)	25.0	3938.0 (3422.0–4156.0)	81.3	0.0121
Magnesium (mg), median (IQR)	267.0 (229.0–319.0)	125.0	490.0 (428.0–535.0)	87.5	0.0006
Calcium (mg), median (IQR)	717.0 (507.0–821.0)	6.3	1161.0 (936.0–1390.0)	37.5	0.0005
Phosphorus (mg), mean (SD)	1311.0 (238.0)	93.8	1627.0 (295.0)	100.0	0.0007
Iron (mg), median (IQR)	10.0 (9.0–11.0)	87.5	17.0 (15.0–21.0)	100.0	0.0012
Zinc (mg), mean (SD)	11.0 (2.0)	31.3	16.0 (3.0)	87.5	<0.0001
Iodine (mg), mean (SD)	88.0 (28.0)	0.0	192.0 (55.0)	81.3	<0.0001

SD, standard deviation; IQR, Interquartile Range (25th–75th centiles); RDI, Recommended Daily Intake; AI, Acceptable Intake; N/A, not applicable * RDI for age and gender or AI for age and gender as appropriate, as per the National Health and Medical Research Council [19]. Statistical significance defined as *p* < 0.05.

**Table 4:** Nutrient biomarkers before and after the weight loss intervention (*n* = 28).

Biomarker (mg/L)	Pre-Intervention (*n* = 28)	Post-Intervention (*n* = 28)	*p*-Value
**Plasma Fatty Acids**			
Total FA, median (IQR)	5181 (4563–5924)	4596 (4106–5166)	0.0070
SFA, median (IQR)	1753 (1518–1954)	1492 (1359–1675)	0.0026
MUFA, median (IQR)	1520 (1338–1747)	1337 (1152–1602)	0.0019
*n*-6 PUFA, median (IQR)	1702 (1430–1816)	1487 (1359–1666)	0.0264
*n*-3 PUFA, median (IQR)	246 (200–281)	246 (199–312)	0.4593
*n*-6:*n*-3 ratio, mean (SD)	6.8 (1.9)	6.2 (2.2)	0.0336
**Plasma Carotenoids**			
Lutein/zeaxanthin, mean (SD)	319 (170)	375 (233)	0.0754
β-cryptoxanthin, median (IQR)	71 (55–95)	78 (56–136)	0.3676
Lycopene, median (IQR)	434 (295–649)	462 (278–752)	0.4349
α-carotene, median (IQR)	14 (0–31)	32 (20–61)	0.0018
β-carotene, median (IQR)	124 (84–206)	149 (119–348)	0.0013
Total carotenoids, median (IQR)	990 (861–1314)	1259 (933–1583)	0.0776

SD, standard deviation; IQR, Interquartile Range (25th–75th centiles); FA, fatty acid; SFA, saturated fatty acid; MUFA, monounsaturated fatty acid; n-6 PUFA, omega-6 polyunsaturated fatty acid; *n*-3 PUFA, omega-3 polyunsaturated fatty acid. Statistical significance defined as *p* < 0.05.

**Table 5:** Multiple linear regression examining baseline eating behaviours, dietary quality, demographics and lung function as predictors of change in % weight.

% Weight Change	Unadjusted Model		Final Model	
			*R*^2^ = 49.3%	*p* = 0.001
Variable (Pre-Intervention)	Coefficient	*p*-Value	Coefficient	*p*-Value
Age (years)	−0.182	0.021	−0.169	0.025
Gender	1.006	0.343		
GOLD Stage	0.274	0.710		
Plasma carotenoid (mg/L)	−0.004	0.008	−0.004	0.009
Plasma SFA (mg/L)	0.002	0.065	0.002	0.078
Uncontrolled eating	−0.151	0.001	−0.149	<0.001
Emotional eating	0.038	0.139	0.045	0.058

R^2^, the coefficient of determination; GOLD, Global initiative for chronic obstructive lung disease; SFA, saturated fatty acid. % weight change calculated as; (post-intervention weight minus pre-intervention weight) divided by pre-intervention weight multiplied by 100. Statistical significance defined as *p* < 0.05.
